# 

**Published:** 1880-02

**Authors:** 


### Table of Contents.

### ORIGINAL COMMUNICATIONS.

Advancement in Gynæcology. By J. G. Westmoreland, M,D , Atlanta, Ga.......... 643
A Case of Ano-Rectal Abscess. By E. A. Cobleigh, M.D , Athens, Tenn.......... 647
Dyspepsia—Its Causes and Treatment. By R. W. Westmoreland, M.D., Atlanta, Ga.......... 652

### REPORTS OF SOCIETIES.

Baltimore Academy of Medicine.......... 654
Proceedings of the Cincinnati Medical Society.......... 660

### SELECTIONS

Vomiting of Pregnancy.......... 667
Diagnosis of Tobacco Amblyopia.......... 669
Select Topics of Modern Surgery.......... 672

### EDITORIAL.

Atlanta Medical College.......... 682
National Board of Health.......... 684
In Memoriam.......... 685
Sixth Decennial Pharmacopœia Convention.......... 688

### BIBLIOGRAPHICAL.

A System of Medicine.......... 689
The Therapeutics of Gynæcology and Obstetrics.......... 689
The Hypodermic Injection of Morphia.......... 690
Paraceutesis of the Pericardium.......... 691
American Health Primer—Brain Work and Overwork.......... 691
Pamphlets Received.......... 691
Meteorological Report for January, 1880.......... 692

### EXCERPTA.

A New Anthelmintic.......... 693
A List of Fraudulent Medical Colleges which sell Diplomas.......... 693
Pilocarpine in Uræmia.......... 694
Treatment of Bursa Patellæ Amplificata.......... 695
For Baldness.......... 696
Puerperal Fever.......... 696
Nitrite of Amyl in Uterine Hemorrhage.......... 697
Bursa Pastoris as Emmenagogue.......... 698
Quinine Test.......... 698
Test for Quinia Sulphate.......... 699
Elegant Process of Capsuling Bottles.......... 700
Application of Plaster-of-Paris Bandages.......... 700
Hepatic Colic.......... 701
Changes of Muscle-Irritability Dependent on the Blood Supply.......... 702
The Benzoates in the Treatment of Tuberculosis.......... 702
How to Grasp the Pelvis for Fixation in Contraction of the Hip.......... 705
Treatment of Asthma by Potassium Iodide.......... 705
Physiological Experiments on a Person Decapitated.......... 706
Formulary.......... 706
Palatable Castor Oil.......... 706

